# Impact of anatomical factors on surgical planning and outcomes in high-risk patients undergoing prophylactic mastectomy

**DOI:** 10.3389/fsurg.2025.1651775

**Published:** 2025-09-24

**Authors:** Tomas Maciulaitis, Daiva Gudavicienė, Nerijus Jakutis

**Affiliations:** Faculty of Medicine, Institute of Clinical Medicine, Clinic of Rheumatology, Orthopaedics Traumatology and Reconstructive Surgery, Vilnius University, Vilnius, Lithuania

**Keywords:** prophylactic mastectomy, nipple-sparing mastectomy, breast reconstruction, preshaping surgery, breast ptosis, implant-based reconstruction

## Abstract

**Background:**

Nipple-sparing mastectomy (NSM) with immediate implant-based reconstruction is widely accepted and effective prophylactic surgical approach for women with high-risk of hereditary BC. However, anatomical factors—advanced breast ptosis and increased sternal notch-to-nipple (SN-N) distance—can increase technical difficulty and complication risk. Preshaping procedures may optimize anatomy and broaden NSM eligibility. This study evaluates the role of preshaping in facilitating safe NSM with implant-based reconstruction.

**Methods:**

We conducted a retrospective analysis of 84 patients who underwent prophylactic mastectomy at Vilnius University Hospital Santaros Klinikos between 2018 and 2024. All had confirmed pathogenic mutations associated with hereditary BC risk. At the time of analysis, 76 patients had completed mastectomy, while 8 had undergone only the preshaping procedure. Patients were divided into two cohorts: single-stage NSM with direct-to-implant reconstruction, and a two-stage approach involving initial preshaping surgery followed by delayed NSM. Anatomical features, surgical timing, complications were analyzed.

**Results:**

Among 76 patients, 63.2% underwent single-stage and 36.8% two-stage reconstruction. Two-stage patients had significantly greater SN-N distances (26.4 ± 3.1 cm vs. 21.6 ± 3.2 cm, *p* < 0.001). The overall complication rate was 7.9%, higher in the single-stage group (10.4%) than the two-stage group (3.6%). In the single-stage cohort, complications correlated with higher ptosis grades (*p* = 0.0021). Ductal carcinoma *in situ* was found in one patient from each group.

**Conclusions:**

Preshaping surgery effectively optimizes anatomy for NSM, reducing complications in patients with ptosis or extended SN-N distances. The two-stage approach offers safe and favorable outcomes in anatomically challenging cases.

## Introduction

Prophylactic mastectomy (PM) is widely recognized as the most effective strategy for preventing breast cancer (BC) in women with a high genetic risk of hereditary BC (1). Among the available surgical techniques, nipple-sparing mastectomy (NSM), combined with immediate implant-based reconstruction, is a widely accepted and effective option in appropriately selected patients. This approach provides reliable oncologic safety while offering superior aesthetic outcomes, thereby improving patient satisfaction, quality of life and psychological benefits (2, 3).

However, NSM via implant-based reconstruction is not suitable for all patients. Factors such as advanced breast ptosis, large breast size, or poor soft tissue quality can increase the technical difficulty and complication risk of immediate reconstruction (4, 5). In these cases, preshaping surgeries are recommended to optimize the breast's anatomical condition, thereby enabling NSM with implants in patients who otherwise would be unsuitable candidates (6). In this study, we use the term preshaping to refer to mastopexy and/or breast reduction procedures performed prior to nipple-sparing mastectomy (NSM) with the goal of optimizing breast anatomy.

The primary aim of this study is to evaluate the role of these preshaping surgeries in facilitating successful NSM with implant reconstruction among high-risk patients. We analyse variations in anatomical parameters across patient groups subjected to different surgical approaches, present demographic characteristics (including age and mutation type), and evaluate oncological safety over time when comparing two-step to one-stage approaches. Additionally, we assess the incidence of postoperative complications.

## Methods

### Study design

This retrospective study includes 84 patients who underwent prophylactic mastectomy or preshaping surgery at Vilnius University Hospital Santaros Klinikos between January 2018 and January 2024. The study was approved by the Vilnius Regional Biomedical Research Ethics Committee (Approval No. 2023/12-1548-1017). At the time of the research, prophylactic mastectomy had been fully completed for 76 patients, while 8 patients had undergone only the preshaping procedure. Inclusion criteria were patients with confirmed genetic mutations associated with a high risk of breast cancer (BC), but without active disease. Exclusion criteria included patients who underwent mastectomy for therapeutic rather than prophylactic reasons.

### Patient population

The study consisted of women aged 20–67 years, all of whom had confirmed pathogenic mutations in BRCA1, BRCA2, or CHEK2. Data on patient demographics, timing of surgeries and consultations, diagnosis, anatomical parameters [such as sternal notch to nipple distance (SN-N), and grade of breast ptosis] were collected and analysed. Breast ptosis was classified according to the Regnault classification system. Complication rates were documented. Only major complications, requiring hospitalization were included in the analysis.

### Surgical techniques

The patients were divided into two groups based on the surgical technique employed: immediate implant-based breast reconstruction and two-stage reconstruction, involving a preshaping surgery followed by a delayed nipple-sparing mastectomy and direct-to-implant reconstruction. Following both the mastectomy and preshaping surgeries, all breast tissues were subjected to a thorough histopathological examination.

### Data collection and statistical analysis

Comprehensive data were collected from electronic medical records, including patient demographics, genetic mutations, grade of ptosis, intraoperative details, and postoperative outcomes. Descriptive statistics were used to summarize patient characteristics. Student's*t*-test was applied for comparisons of quantitative variables with equal variances, and Welch's *t*-test was applied when variances were unequal. Fisher's exact test was used for categorical data when *n* ≤ 5. Statistical significance was defined as *p* < 0.05. In addition, a *post-hoc* power analysis was performed for key subgroup comparisons (complication rates by surgical approach and by ptosis grade) using a two-sample proportion test (Cohen's *h*) at *α* = 0.05 (two-sided).

## Results

### Patient demographics and genetic characteristics

The study consisted of 84 women with a mean age of 43.8 years (SD ± 9.2). The mean body mass index (BMI) of the entire cohort was 24.8 kg/m^2^ (SD ± 3.9), ranging from 18.2 to 33.6 kg/m^2^. There was no statistically significant difference in BMI between patients who underwent single-stage vs. two-stage reconstruction (*p* = 0.41).

The distribution of genetic mutations is detailed in Table 1. BRCA1 mutations were predominant, accounting for 76.62% of cases, while BRCA2 and CHEK2 mutations were present in 25% and 2.38% of patients, respectively. This distribution aligns with known epidemiological trends in the Eastern European region.

**Table 1:** Genetic mutations and breast cancer history.

Mutation type	Prior BC history	No prior BC history	Total (*N*, %)
BRCA1	30 (69.77%)	31 (75.61%)	61 (76.62%)
BRCA2	11 (25.58%)	10 (24.39%)	21 (25.00%)
CHEK2	2 (4.65%)	0 (0.00%)	2 (2.38%)

### Surgical techniques

**Figure 1:**
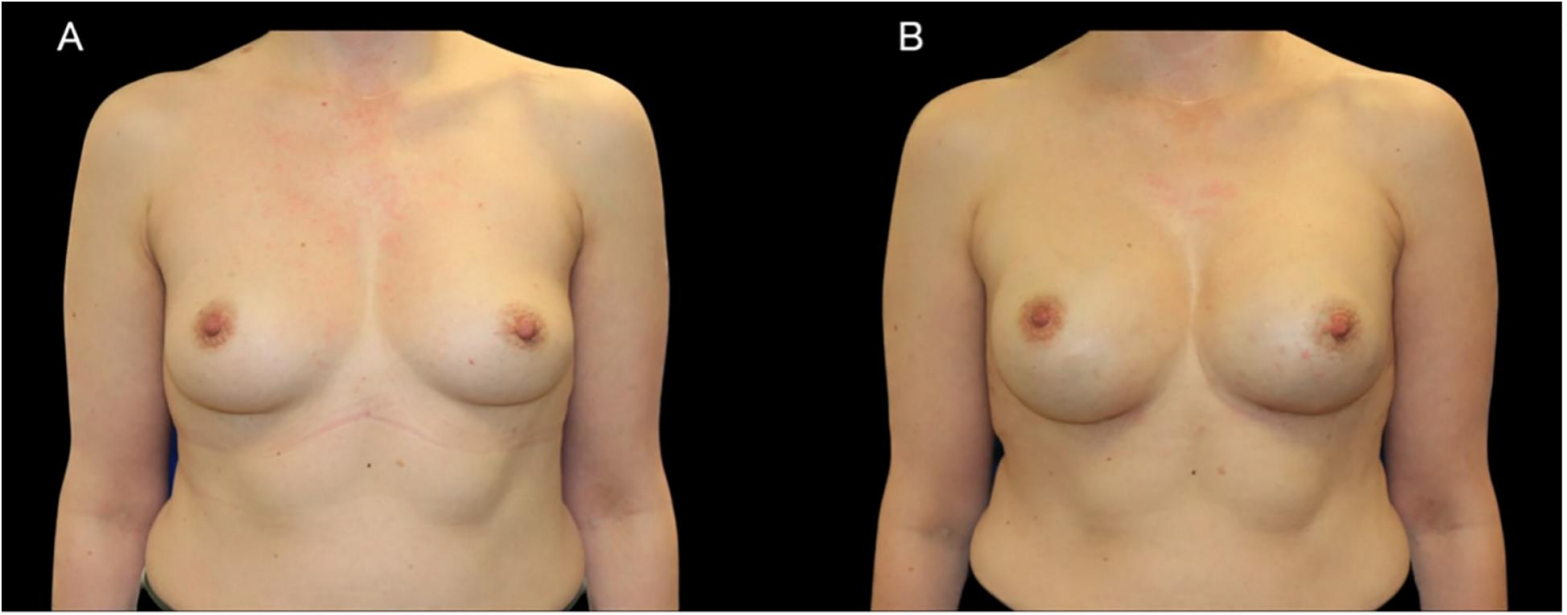
A case of 44-year-old woman with a BRCA1 pathogenic variant who underwent bilateral NSM with immediate subpectoral implant-based reconstruction using 345 cc anatomic implants. **(A)** Preoperative photograph. **(B)** Postoperative result.

**Figure 2:**
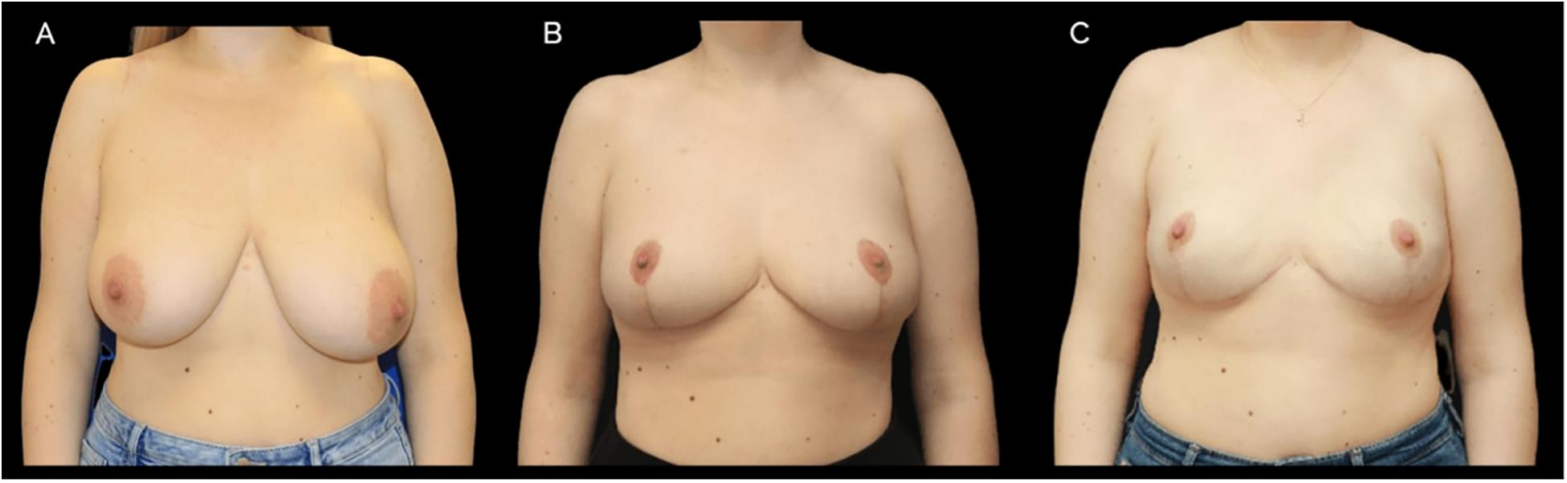
A case of 42-year-old female with BRCA1 genetic mutation. Initially, a preshaping breast reduction was performed, followed by risk reducing bilateral nipple-sparing mastectomy (NSM) and immediate implant-based reconstruction 4 months later. Prepectoral 450 cc round implants were used with a surgical mesh. **(A)** Preoperative photograph **(B)** postoperative image after preshaping, 4 months following surgery. **(C)** Final result after implant-based reconstruction.

Figures 1, 2 show representative patient photographs from each surgical group. A significant proportion of the patients, 63.16% (*n* = 48), underwent single-stage reconstruction with direct-to-implant placement. The remaining 36.84% (*n* = 28) underwent a two-stage approach, involving a preshaping surgery followed by a delayed nipple-sparing mastectomy and direct-to-implant reconstruction.

Implant volume data were available for all patients. In the single-stage group (*n* = 48), the mean implant volume was 319.4 ± 68.5 cc (range: 180–545 cc). In the two-stage group (*n* = 28), the mean implant volume was 361.1 ± 66.1 cc (range: 225–530 cc). All implants used were Mentor® silicone gel–filled devices.

### Anatomical considerations

The choice of surgical method was strongly influenced by anatomical factors, particularly the degree of breast ptosis and the sternal notch-to-nipple distance.

Single-stage surgery was most frequently chosen for patients with no breast ptosis 50% (*n* = 24), first-grade ptosis 29, 17% (*n* = 14), and second-grade ptosis 20, 83% (*n* = 10).

The preshaping surgery method was primarily used for patients with second-degree breast ptosis 67, 86% (*n* = 19), followed by third-grade breast ptosis 21, 43% (*n* = 6) and first-grade breast ptosis 10, 71% (*n* = 3).

**Figure 3:**
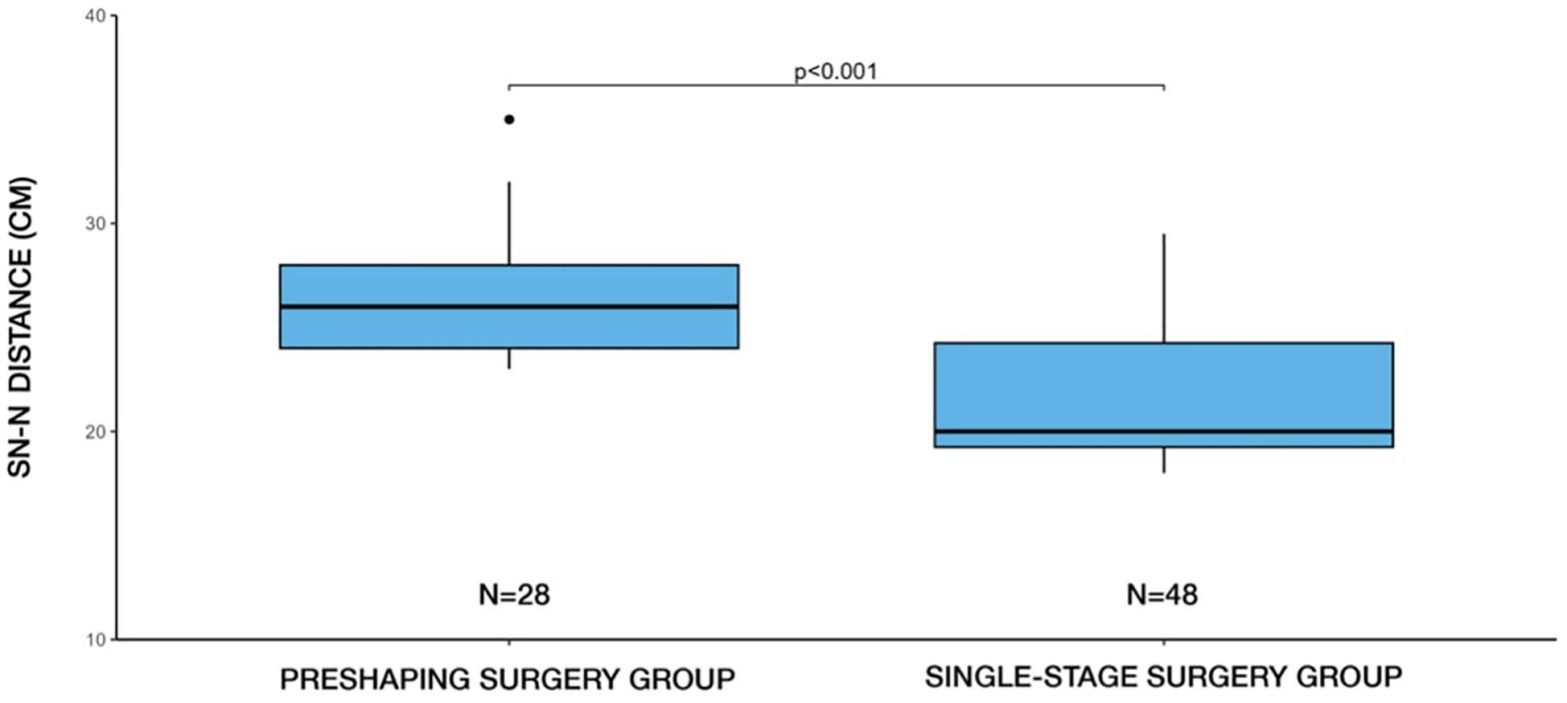
Distribution of surgical methods (single-stage vs. two-stage) by SN-N distance. Two-stage (preshaping) surgery was more frequently selected in patients with longer SN-N distances, whereas single-stage surgery predominated in cases with shorter SN-N distances.

The mean preoperative sternal notch-to-nipple (SN-N) distance for patients undergoing single-stage surgery was 21.6 cm (±3.21), with a minimum of 18 cm and maximum of 29.5 cm. In contrast, for patients undergoing two-stage surgery, the mean SN-N distance was 26.4 cm (±3.12), with a minimum of 23 cm and a maximum of 35 cm. A statistically significant difference was found (*p* < 0.001, Student's t-test) between the surgical approach method and the SN-N distance (Figure 3).

In our clinical practice, a sternal notch-to-nipple (SN–N) distance exceeding 27 cm**,** particularly when accompanied by Regnault grade II or III ptosis, generally guided us toward recommending a two-stage approach. Additional factors such as breast volume, anticipated implant size, and soft tissue quality were also taken into account. In borderline cases, the final decision was made through shared decision-making with the patient, balancing the desire to minimize the number of surgeries against the potential benefits of a staged approach for safety and aesthetic outcome.

### Postoperative complication rates

Postoperative complications were observed in 7.9% (*n* = 6) of the study population, with a higher incidence observed in the single-stage reconstruction group (*n* = 5) compared to the two-stage group (*n* = 1). No statistically significant association was found between the frequency of postoperative complications and the chosen surgical method (*p* > 0.05).

In the single-stage group (*n* = 48), three patients (6.25%) developed complications related to partial mastectomy flap necrosis leading to implant exposure and requiring implant salvage procedures. One patient (2.08%) experienced early postoperative implant displacement requiring reoperation, and one patient (2.08%) developed a wound infection treated successfully with IV antibiotics. Among patients who underwent the preshaping surgery, one patient (3.57%) developed a skin flap compromise that progressed to implant infection and was successfully managed with implant salvage procedure.

Complications were more frequent among patients with higher grades of breast ptosis undergoing single-stage procedures. A statistically significant association was found between higher ptosis grade and increased complication rates in single-stage procedures (*p* = 0.0021) (Table 2).

**Table 2:** Complication rates by ptosis grade and surgical approach.

Surgical approach	Ptosis grade	No complication (*n*)	Complication (*n*)
Single stage (*n* = 48)	No ptosis	24 (100.0%)	0 (0.00%)
	Grade I	13 (92.86%)	1 (7.14%)
	Grade II	6 (60.00%)	4 (40.00%)
Two-stage (*n* = 28)	Grade I	3 (100.00%)	0 (0.00%)
	Grade II	18 (100.00%)	0 (0.00%)
	Grade III	6 (85.71%)	1 (14.29%)

*Post-hoc* power analysis indicated that the comparison of complication rates between single-stage (10.4%, 5/48) and two-stage (3.6%, 1/28) groups corresponded to a small effect size (*h* = 0.277) with low achieved power (∼21%). In contrast, within the single-stage cohort, the difference between Grade II ptosis (40.0%, 4/10) and no/Grade I ptosis (2.6%, 1/38) reflected a large effect (*h* = 1.044) with adequate power (∼84%).

The mean clinical follow-up duration was 14.2 ± 3.1 months in the single-stage group and 15.0 ± 3.4 months in the two-stage group (*p* = 0.38). While follow-up completeness varied slightly among patients—as is common in retrospective studies—this timeframe was sufficient to capture early postoperative complications, which were the primary outcomes of interest.

### Timing of surgery

The average interval between the preshaping procedure and subsequent mastectomy was 268.96 days (SD ± 159.55).

The mean time from initial consultation—following referral by a geneticist after confirmed mutation diagnosis—to the mastectomy stage was significantly longer in the preshaping group (mean: 384 days, SD: 164) compared to the single-stage group (mean: 136 days, SD: 93.2). This difference was statistically significant (*p* < 0.001).

All patients undergoing a staged approach remained under interim high-risk surveillance during the interval between preshaping and mastectomy, including clinical examination with breast ultrasound every 6 months and annual breast MRI. No interval cancers were identified in this cohort prior to definitive mastectomy.

### Resected tissue volume

In the two-stage cohort, the mean volume of tissue removed during the initial preshaping procedure was 217.1 ± 160.4 g per breast (*n* = 56 breasts), while the second-stage mastectomy yielded an average resection weight of 366.0 ± 155.5 g per breast (*n* = 56 breasts).

In comparison, patients in the single-stage group had a mean resection volume of 249.7 ± 175.9 g per breast at the time of mastectomy (*n* = 96 breasts).

These findings support the clinical observation that patients selected for a staged approach typically present with greater breast volume and more advanced ptosis, necessitating preparatory reshaping to optimize outcomes.

### Histopathological findings

Among the 48 patients who underwent a single-stage surgery, malignancy changes were identified in the excised tissue of one patient (2.1%). In the two-stage group (*n* = 28), no malignancies were detected following the initial preshaping stage. However, after the second stage (mastectomy and reconstruction), malignancy changes were found in one patient (3.6%).

## Discussion

This comprehensive analysis provides critical insights into surgical planning for high-risk patients undergoing PM with implant-based reconstruction. Our findings emphasize the significance of anatomical factors—particularly breast ptosis and SN-N distance—in determining surgical strategy. Patients with advanced ptosis or longer SN-N distances were more frequently selected for staged approach, which is consistent with the literature advocating preshaping procedures in anatomically challenging cases.

The interval between preshaping and final mastectomy with reconstruction plays a crucial role in oncological and functional safety of this exact approach. In our cohort, the average interval of approximately 9 months between these procedures aligns well with published literature. For example, Gunnarsson et al. recommend a 4-moths delay, while Alperovich et al. suggest at least one year ^(^7, 8). Barnes et al. reported an average interval of 213 days (∼7 months) for PM after preshaping procedures ^(^9). It is important to highlight that the study was conducted during the period of COVID-19 pandemic, which may have impacted the time intervals between surgeries, particularly in the two-stage.

The extended interval inherent to a staged approach raises a theoretical oncologic concern for mutation carriers, as it prolongs the window during which a malignancy could develop. In our institution, this risk was mitigated by interim surveillance consisting of regular clinical examination, breast ultrasound every 6 months, and MRI once a year. After prophylactic mastectomy, patients continued surveillance with annual ultrasound, and MRI was performed if clinically indicated. Importantly, no interval cancers were identified in our staged group.

The primary concern with performing both reduction of soft tissues and mastectomy simultaneously is the disruption of blood flow to the skin flap and NAC, which increases the risk of necrosis of the NAC (5). A two-stage approach allows for revascularization and remodeling of tissue, thus preserving perfusion and reducing breast skin flap necrosis risk. While overall complication rates did not differ significantly between groups in our cohort, the finding that higher ptosis grades were associated with increased complications only in the single-stage group suggests that preshaping approach may reduce this specific risk.

Importantly, oncological safety was preserved across both approaches. Cancerous changes were identified in only one patient in each group (2.1% in the single-stage and 3.6% in the two-stage group), with no evidence of increased risk of recurrence (*p* > 0.99). Both identified cases were ductal carcinoma *in situ* (DCIS). However, the small number of patients in each subgroup limits the strength of these findings and precludes definitive conclusions. Larger, prospective, and multicentric studies are needed to validate the oncological safety of the preshaping approach.

Furthermore, the study by Choi et al. underscores the critical role of sternal notch-to-nipple (SN–N) distance in surgical planning for nipple-sparing mastectomy (NSM) (10). The authors identified an SN–N distance greater than 27 cm as a significant predictor of complications in patients undergoing immediate implant-based reconstruction without prior anatomical modification. Based on these findings, they advocate for preshaping procedures to optimize breast geometry and improve surgical outcomes before NSM.

Our findings are consistent with these observations. We observed a statistically significant difference in SN–N distance between patients undergoing single-stage and two-stage reconstruction (*p* < 0.001). In our cohort, the mean SN–N distance was 21.6 cm (±3.21) in the single-stage group and 26.4 cm (±3.12) in the two-stage group. Although no strict threshold was predefined, this pattern suggests that SN–N distance is a relevant anatomical factor influencing the decision to pursue a staged approach. In our clinical experience, a distance exceeding 27 cm**,** particularly in combination with moderate-to-severe ptosis, typically prompts consideration of a two-stage strategy to optimize nipple positioning and reduce complication risk.

**Figure 4:**
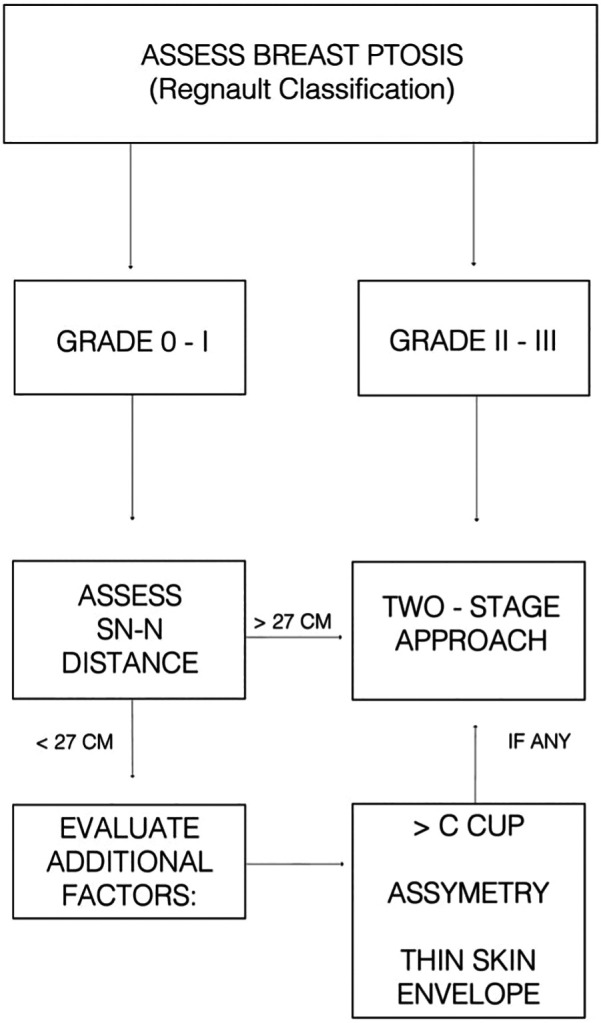
Suggested clinical algorithm to guide decision-making between single-stage and two-stage NSM with implant-based reconstruction.

Based on our institutional experience and supporting literature (10), we propose a simplified decision-making algorithm (Figure 4) for selecting patients for single-stage vs. two-stage NSM. This framework incorporates key anatomical and patient-specific factors while allowing for individualized treatment planning. In our study, preshaping was used selectively in patients with advanced ptosis or extended SN–N distance, and is not intended for use in non-ptotic cases.

Postoperative complication rates were low overall (9.2%) but were notably higher in patients with advanced ptosis undergoing single stage reconstruction. This finding suggests that while immediate reconstructive surgeries offer the benefit of reduced overall surgical time, they may pose a higher risk for complications in certain patient subsets. Future studies should explore strategies to mitigate these risks, such as enhanced surgical techniques or patient-specific preoperative interventions. It is important to emphasize that risk-reducing mastectomy itself carries an inherent risk of perioperative complications, independent of anatomical modifications such as preshaping. Even in well-selected patients, a certain baseline complication rate is to be expected due to the complexity of the procedure.

This study is limited by its retrospective nature and the relatively small sample size, particularly in the subgroup analyses. *Post-hoc* power analysis confirmed that the comparison of complication rates between single-stage and two-stage groups was underpowered (∼21% power for the observed difference). These findings should therefore be interpreted as hypothesis-generating rather than definitive. By contrast, the association between Grade II ptosis and increased complications in the single-stage cohort had adequate power (∼84%), supporting this specific observation.

Furthermore, while the current follow-up was sufficient to capture early complications, it is not adequate to assess long-term oncological safety or durability of reconstruction. At our institution, all high-risk patients are enrolled in long-term surveillance programs that include regular clinical examination and ultrasound of the reconstructed breasts. Extended follow-up (5–10 years) is ongoing, and future analyses will provide more comprehensive oncological and reconstructive outcome data. Larger prospective studies are required to validate these findings and refine surgical decision-making algorithms.

## Conclusions

This five-year retrospective analysis highlights the importance of individualized surgical planning in high-risk patients undergoing PM with implant-based reconstruction. Our findings indicate that preshaping procedures play a valuable role in optimizing the breast's anatomical condition, thereby expanding the cohort of patients who can successfully undergo NSM with immediate reconstruction, particularly those initially seemed unsuitable due to factors like significant ptosis or large breast volume.

## Data Availability

The original contributions presented in the study are included in the article/Supplementary Material, further inquiries can be directed to the corresponding author.
